# Postural development in school children: a cross-sectional study

**DOI:** 10.1186/1746-1340-15-1

**Published:** 2007-01-04

**Authors:** Danik Lafond, Martin Descarreaux, Martin C Normand, Deed E Harrison

**Affiliations:** 1 Département des Sciences de l'activité physique, Université du Québec à Trois-Rivières, 3351, boul. des Forges, C.P. 500, Trois-Rivières (QC), G9A 5H7, Canada; 2 Département de Chiropratique, Université du Québec à Trois-Rivières, 3351, boul. des Forges, C.P. 500, Trois-Rivières (QC), G9A 5H7, Canada; 3 Ruby Mountain Chiropractic Center & CBP NonProfit Inc, Elko, NV 89801, USA

## Abstract

**Background:**

Little information on quantitative sagittal plane postural alignment and evolution in children exists. The objectives of this study are to document the evolution of upright, static, sagittal posture in children and to identify possible critical phases of postural evolution (maturation).

**Methods:**

A total of 1084 children (aged 4–12 years) received a sagittal postural evaluation with the Biotonix postural analysis system. Data were retrieved from the Biotonix internet database. Children were stratified and analyzed by years of age with n = 36 in the youngest age group (4 years) and n = 184 in the oldest age group (12 years). Children were analyzed in the neutral upright posture. Variables measured were sagittal translation distances in millimeters of: the knee relative to the tarsal joint, pelvis relative to the tarsal joint, shoulder relative to the tarsal joint, and head relative to the tarsal joint. A two-way factorial ANOVA was used to test for age and gender effects on posture, while polynomial trend analyses were used to test for increased postural displacements with years of age.

**Results:**

Two-way ANOVA yielded a significant main effect of age for all 4 sagittal postural variables and gender for all variables except head translation. No age × gender interaction was found. Polynomial trend analyses showed a significant linear association between child age and all four postural variables: anterior head translation (p < 0.001), anterior shoulder translation (p < 0.001), anterior pelvic translation (p < 0.001), anterior knee translation (p < 0.001). Between the ages of 11 and 12 years, for anterior knee translation, T-test post hoc analysis revealed only one significant rough break in the continuity of the age related trend.

**Conclusion:**

A significant linear trend for increasing sagittal plane postural translations of the head, thorax, pelvis, and knee was found as children age from 4 years to 12 years. These postural translations provide preliminary normative data for the alignment of a child's sagittal plane posture.

## Background

The lifetime prevalence of low back pain among schoolchildren ranges from 20% to 51% [1-4]. Recent literature reviews indicate that back pain in children can be correlated to several risk factors such as prolonged sitting posture, faulty spinal posture, and abdominal muscles weakness [2,5]. It has also been suggested that discrepancies between childhood anthropometric characteristics and school furniture dimension could be responsible for the development of musculoskeletal conditions [6,7]. In fact, prolonged sitting postures and school bag carriage are equally associated with back pain [8].

Because back pain during childhood and adolescence is known to be an important predisposing factor for experiencing back pain into adulthood [9,10], prevention of and screening for risk factors of back pain in childhood may be important. For children and adolescents, upright posture measurements might be a useful clinical tool to identify and prevent the developmental process of musculoskeletal conditions in its early stages. For instance, measurements of acute spinal postural changes associated with load carriage have been used to experimentally approximate the potential risk to induce back pain [11]. In this context, postural analysis is aimed at identifying abnormal deviation from a referenced vertical alignment (plumb line) in the frontal and sagittal planes [12]. A vertical segmental alignment close to the ideal reference posture is commonly considered to be a measure of good musculoskeletal health. However, the assumption that faulty posture developed during childhood can lead to future back pain lacks scientific evidence. There is also a need for rigorous normal reference data in school-aged children.

As they grow and age, children's posture may change considerably. For example, segmental sagittal plane analysis, on children and adolescents, has recently been performed using radiographyto document the normal evolution of the sagittal alignment with growth [13-17].

Gilliam *et al*. consider radiology to be the most accurate method to assess static positioning using bony landmarks [18]. However, clinical assessment of postural alignment based on non invasive techniques, such as postural video analysis, have the advantages of being less expensive and more appropriate for screening evaluations. Furthermore, these techniques do not expose individuals to ionising radiation. This may prove important, particularly when postural alignment has to be evaluated in pregnant women, disabled or young populations.

In a clinical setting, contemporary postural analysis systems enable the clinician to rapidly perform a quantitative postural evaluation and could eventually be used in patient counselling and treatment monitoring. Several such systems have been found to have high degrees of reliability and validity and are easy to use in a clinical setting [19-23].

Postural screening and evaluation protocols in the primary and secondary prevention of musculoskeletal conditions are still evolving. In particular, clinically relevant non-invasive data about critical phases of postural development in schoolchildren are lacking.

The aim of this cross-sectional study was to document the evolution of upright, static, sagittal posture in children aged between 4–12 years old and to identify possible critical phases of postural evolution (maturation). The main hypothesis of this study was that, children's posture will gradually deviate from the ideal sagittal postural alignment with maturation, between the ages of 4 to 12 years old.

## Methods

A total of one thousand eighty four (1084) postural analyses of children between the ages of 4 years to 12 years were performed with subjects using the web based system from Biotonix™ [24]. All postural analysis data were obtained from the Biotonix™ database and were gathered from several chiropractic and physical therapy clinics between the months of June 2001 to October 2004. All pediatric datafiles in this date and age range were accessed and analyzed for the current study. All 1084 of these children presented for 'postural screening' analysis or presented for evaluation of various musculo-skeletal complaints. The Ethics Committee of the Université du Québec à Trois-Rivières gave approval for this study.

BioTonix™ offers a program, termed the BioPrint^® ^computer system, for postural analysis. BioPrint^® ^requires a set of 3 photographs of each subject: 1) a right lateral, 2) an antero-posterior, and 3) a posterior – anterior view. Subjects stand 22.9 cm from the center of a calibrated wall grid and the photographs are obtained with a digital camera. The camera height is at 83.8 cm above the floor and the camera is placed between 2.44 m to 3.35 m (according to room space) from the wall grid on a perpendicular line from mid-wall grid.

For the BioPrint^® ^evaluation, the subjects were asked to wear tight fitting clothes in order for examiners to find various anatomical sites. According to the system requirements, the examiners placed a series of 26 flat markers and six white sphere markers on each subject before taking the 3 photographs. For the photographic procedures, subjects were instructed to stand, nod their head up and down twice with their eyes closed and then assume what they felt to be a neutral body posture. These procedures for postural analysis have been found to be reliable [25]. In order to identify and quantify sagittal plane translations six anatomical sites with reflectors are used: 1) the tragus, 2) the acromion, 3) the antero-superior iliac spine, 4) the postero-superior iliac spine, 5) the fibular head, and 6) the fifth metatarsal tuberosity.

**Figure 1:**
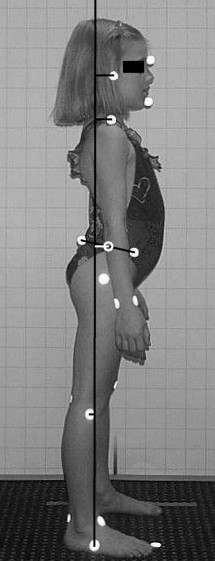
BioPrint Sagittal Picture identifying postural displacement variables.

In the BioPrint^®^, a complete postural profile of the subject is defined by 37 dependent variables that are primarily related to translations and rotations of the head, thorax and pelvis in the frontal and sagittal planes. For this study however, only data from the sagittal plane were analyzed. Eight dependent variables were compared using one way analysis of variance (ANOVA). The angular variables are the angles calculated between points located at: (1) the external auditory meatus on the head and the acromio-clavicular (AC) joint on the shoulder, (2) the AC-joint and the mid pelvis, (3) the mid pelvis (hip joint) and the mid knee, (4) the mid knee and the tarsal joint of the foot. The millimetric distance (translation displacement relative to the tarsal joint) variables are: (1) head, (2) shoulder, (3) pelvis and (4) knee. Figure 1 depicts the translation displacement variables.

### Statistical analysis

All dependent variables were found to be distributed normally and were therefore, submitted to a two-way factorial ANOVA using STATISTICA software (Statsoft, OK, USA). This analysis tested for the main effect of age, the main effect of gender, and the possible age × gender interaction. Predefined polynomial trend analyses were used to test the statistical significance of our *a priori *hypotheses of a gradual postural modification throughout school years. Statistical significance level was set at p < 0.05. After removing the linear trend from the means, a T-test, based on standard error of the mean with Bonferroni corrections (nine comparisons, p < 0.006), was used to compare consecutive year groups in order to identify any significant break in continuity of the postural evolution.

## Results

Subjects' characteristics are presented in Table 1. The two-way ANOVA yielded a significant main effect of age for all dependant variables and a significant main effect of gender for all variables except sagittal head translation. No significant age × gender interaction was noted. Consequently, all gender data were pooled for each age group for the subsequent polynomial trend analyses and T-test post hoc analyses.

**Table 1:** Subjects characteristics

**Age **(years)	**n**	**Male**	**Female**	**Height **(cm)	**Weight **(kg)
4	36	14	22	107.4 ± 10.3	17.8 ± 2.8
5	71	28	43	111.4 ± 11.3	20.6 ± 4.3
6	89	40	49	119.4 ± 12.2	25.1 ± 7.5
7	131	83	48	125.2 ± 10.8	27.6 ± 8.8
8	136	80	56	133.5 ± 12.8	32.6 ± 11.1
9	126	65	61	134.2 ± 8.4	33.0 ± 9.2
10	133	75	58	142.6 ± 9.7	37.2 ± 9.4
11	178	74	104	148.3 ± 21.5	43.0 ± 12.3
12	184	93	91	149.4 ± 42.0	47.2 ± 11.2

Mean ± Standard deviation

**Figure 2:**
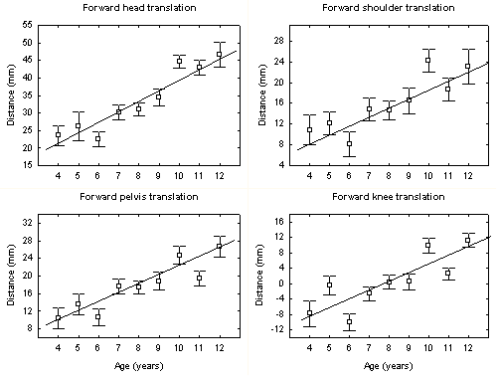
Average values and the standard errors of head, shoulder, pelvis and knee translation displacement variables with respect to age.

Figure 2 shows the average values and the standard deviations for the head, shoulder, pelvis, and knee sagittal translation displacement variables with respect to age. The polynomial trend analyses showed a significant linear association between subject age and all four displacement variables. Statistically significant associations with age were found for forward head translation (F = 49.72, df = (1,1075), *p *< 0.001), forward shoulder translation (F = 15.16, df = (1,1075), *p *< 0.001), forward pelvis translation (F = 29.82, df = (1,1075), *p *< 0.001) and forward knee translation (F = 13.75, df = (1,1075), *p *< 0.001). With the exception of the forward knee translation, which significantly increased between age 11 and 12, T-test post hoc analyses failed to reveal any significant break in the continuity of the trends.

## Discussion

Sagittal plane postural alignment is thought to be important in the risk and development of spinal deformities and pain syndromes. However, little information on quantitative sagittal plane postural alignment and evolution exists in children. This study shows that postural alignment of children, relative to a vertical reference, changes considerably between the ages of 4 to 12 years. Our results show that the postural evolution during childhood is characterized by an increase in forward translation displacements of the head, shoulders, pelvis and knees in the sagittal plane. Our findings are similar to the study by Mac-Thiong *et al*, where radiographic sagittal posture was found to adjust with age; most likely to avoid inadequate anterior displacement of the body center of gravity [13]. In the current study, the finding of forward displacement of the head, shoulder, and pelvis must be coupled with rearward displacement of the center of mass of the thorax in order to maintain an adequate sagittal balance. However, the Bioprint program does not attempt an analysis of the sagittal plane alignment of the center of the thorax.

The current investigation has presented data on the evolution in children of relative sagittal plane translations of the head, shoulders, hip, and knee. The majority of previous investigations have presented data concerning radiographic or surface contour development of the sagittal plane spinal curvature, lumbar lordosis and thoracic kyphosis, during childhood and adolescence [13,14,16,17]. For examples, Poussa *et al*, studied the development of spinal posture in a cohort of 1060 children from the age of 11 to 22 years [14]. Their data indicated that thoracic kyphosis was more prominent in males at all ages and that it increased with age in men but not in women. They also observed a greater lumbar lordosis in women at all ages [14]. In a longitudinal study, Widhe monitored the spinal mobility and sagittal configuration of 90 children at age 5–6 years old and age 15–16 [16]. This study showed that thoracic kyphosis and lumbar lordosis increased between 5 and 16 years old while spinal mobility decreased [16]. In a radiographic study Cil *et al *noted an increase of the lumbar lordosis, from 44°to 57°, in children aged between 3 and 12 years old and then a decrease from ages 13–15 [17]. Oppositely, the thoracic kyphosis increased until the age of 10, decreased between the ages of 10–12 years, and then increased from 13–15 years where the kyphosis approximated the lumbar lordosis [17].

Only a few studies have described the sagittal plane postural alignment profile of the head, thorax, and pelvis alignment of children [22,26,27]. In a plumb line analysis of 144 children aged 6 to 17 years, Ihme *et al *qualitatively assessed the gravity perpendicular alignment of the shoulder center, the greater trochanter, and the lateral ankle [27]. Sagittal postural alignment did not differ in the age groups, but the shoulder center moved anterior with increasing postural insufficiency. The mid pelvic point of children was found to be anteriorly located in healthy children compared to those with a postural insufficiency [27].

Using sagittal plane photographs for upright standing posture of 38 boys and girls aged 5–12 years, McEvoy et al measured five postural angles (trunk, neck, gaze, head on neck, lower limb) [22]. Similar to the results of the current study, McEvoy et al found that the postural angles of the trunk, neck, and lower limb were significantly influenced by age [22]. However, no gender influence on any angle was found. In a study of 294 8–16 years old boys and girls, divided into five age groups, Mellin et al found the upper thoracic sagittal alignment was more vertical among girls [26].

To our knowledge, no previous investigations have looked at the age related evolution of sagittal plane translations of the head, shoulder, and pelvis in children as in the current study.

The sagittal plane postural evolution between age 4 and 12 found in this study can lead to different interpretations. One could suggest that the observed postural modifications are the result of normal musculoskeletal maturation throughout childhood and puberty. Indeed, it could reflect an adaptation process aimed at maintaining an adequate sagittal balance and appropriate configuration in terms of musculoskeletal loads and sagittal plane curvature development [13,17]. On the other hand, several authors have suggested that postural habits and other environmental factors could influence postural development [28,29]. These observations are not surprising since children, attending traditional school, spend over 95% of their school time in a static sitting position [29,30]. Moreover, children and adolescents spend an average of 1.5 hours a day playing video games and using computers [31]. Thus, with the increasing number of hours spent in the sitting position at home and at school during childhood, sagittal plane postural translations may increase with age. Furthermore, this large sample of undiagnosed subjects, mainly from the chiropractic paediatric population, may include several types of disorders that could have affected the results, for instance, Scheuermann's disease. Scheuermann's disease is characterised by an increase thoraco-lumbar kyphosis with compensatory lumbar and cervical lordosis and the incidence of Scheuermann's disease has been estimated at 1–8%, with the most severe presentation commonly appearing between age 12 and 16 years [32]. Therefore, it is possible that Scheuermann's disease could have minor effects on the results of this study, particularly because our sample age was between 4 to 12 years.

There are several limitations to the current investigation. First, the recruited subjects were all undiagnosed. However, they can be considered to be representative of paediatric populations that present to chiropractic and physical therapy clinics. It is possible that some of these patients will develop a spinal disorder in older age and only a longitudinal study can identify this to be correct or incorrect. Second, findings of this cross-sectional study can not define the evolution of the sagittal postural alignment during the childhood between 4 to 12 years old in a given subject. However, in contrast to radiological procedures, the current non-invasive method for postural quantification should allow the selection of children in a longitudinal study to accurately define the association between age and postural variables.

## Conclusion

The current study has supported the hypothesis of larger postural segmental (head, shoulder, pelvis, knee) displacement from the vertical reference in children as they grow and age. It is possible that musculoskeletal conditions such as back pain and neck pain will result in children should the threshold of a tolerable postural displacement be reached. However, this 'tolerable threshold' needs to be determined and investigated in longitudinal studies. If indeed postural abnormalities are associated with increased risk of back pain, the current study results should aid in the research of treatment interventions to prevent or slow sagittal plane postural abnormalities. For instance, these results may be used as normative values of chiropractic paediatric populations to estimate the statistical power (N) needed for further longitudinal studies.

## Competing interests

The author(s) declare that they have no competing interests.

## Authors' contributions

MCN initiated the study and gathered the data. DL and MD handled the data analysis. DL, MD and DEH contributed to study design and wrote the first manuscript draft. All authors read and approved the final manuscript.
